# Aquagenic wrinkling of the palms: review of the literature

**DOI:** 10.1111/ced.15323

**Published:** 2022-09-01

**Authors:** Karen Yang, Cici Zhou, Janiene Luke

**Affiliations:** ^1^ School of Medicine University of California Riverside CA USA; ^2^ Department of Dermatology Loma Linda University Loma Linda CA USA

## Abstract

Aquagenic wrinkling of the palms (AWP) is a rare dermatological disease characterized by development of rapid and excessive wrinkling and oedema of the palms and transient whitish or yellowish papules without erythema on the palmar surfaces after immersion in water. This phenomenon can be accompanied by pain and/or pruritus. The most common treatment of AWP involves aluminium‐based topicals. This article discusses the associations, pathological mechanisms and treatment options of AWP.

## Graphical abstract

Aquagenic wrinkling of the palms (AWP) is a rare dermatological disease characterized by development of rapid and excessive wrinkling and oedema of the palms, and transient whitish or yellowish papules without erythema on the palmar surfaces after immersion in water. This phenomenon can be accompanied by pain and/or pruritus, and the most common treatment of AWP involves aluminium‐based topicals. This article discusses the associations, physiological mechanisms and treatment options of AWP.

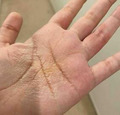

Click here for the corresponding questions to this CME article.

## Introduction

Aquagenic wrinkling of the palms (AWP), also known as aquagenic palmoplantar keratoderma or aquagenic syringeal acrokeratoderma, is a dermatological rare phenomenon characterized by transient oedematous white or translucent papules without erythema on the palmar surfaces after contact with water. AWP affects mostly female patients and the majority of AWP occurs in patients with cystic fibrosis (CF) or CF carriers, although it is also observed in patients with hyperhidrosis, Raynaud disease (RD), marasmus and atopic dermatitis (AD), and in people taking certain medications such as aspirin and rofecoxib.^1^, ^2^, ^3^ In addition, this rare dermatosis can be idiopathic.

## Clinical features

**Figure 1:**
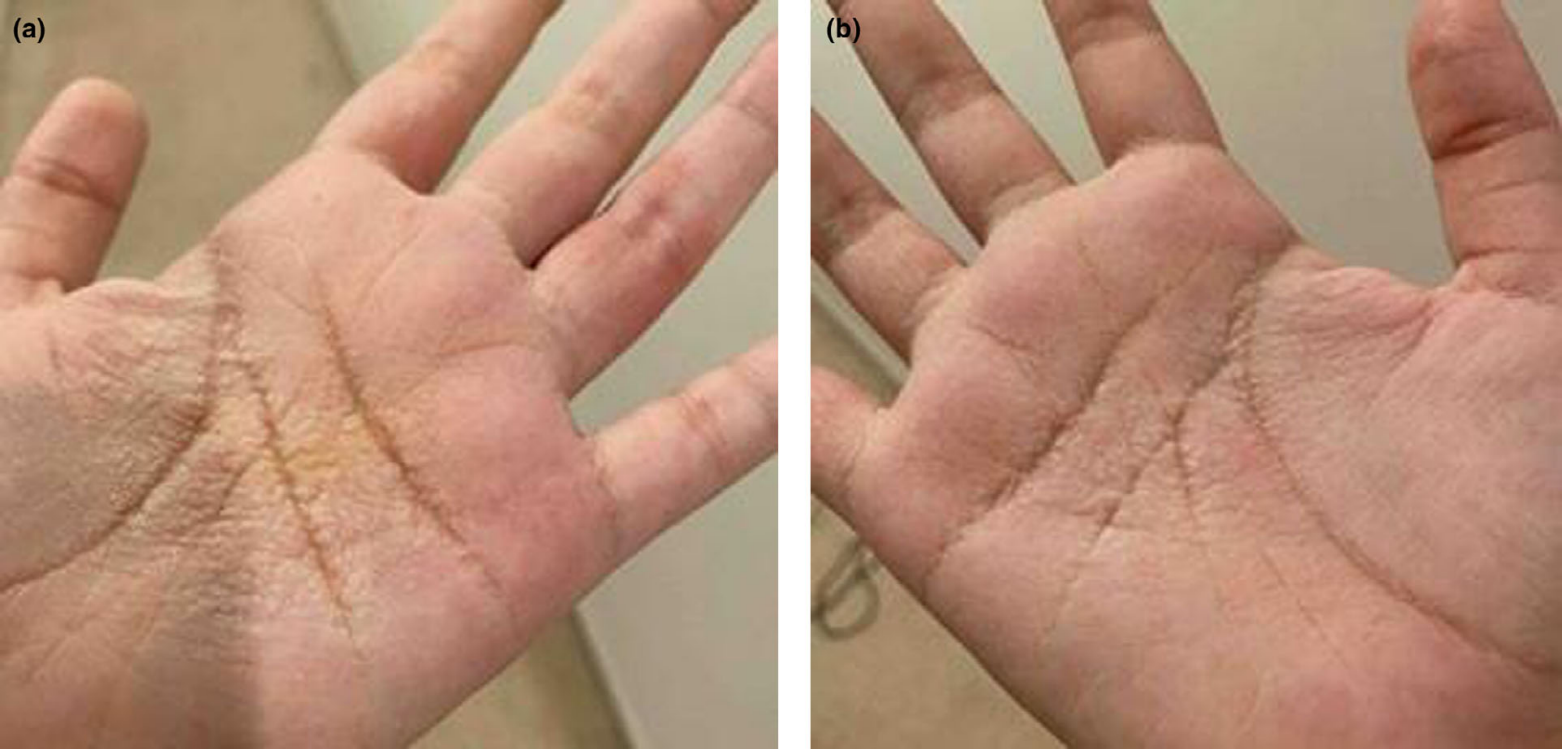
(a,b) Exaggerated wrinkling of palm with keratotic, pebbly, white plaques and prominent eccrine ostia. [Colour figure can be viewed at wileyonlinelibrary.com]

AWP is characterized by the excessive wrinkling and the development of oedematous translucent, yellow or whitish plaques and papules on the palms after exposure to water.^4^ Normal pathological wrinkling of palmar skin occurs after 11 min of water immersion, whereas AWP usually occurs within 3 min of water immersion^5^ and may be accompanied by pain and/or pruritus.^6^ An example of the exaggerated wrinkling in both palms in AWP, along with keratotic, pebbly, white plaques and prominent eccrine ostia is shown in Fig. 1a,b. The rapid development of these skin lesions after water exposure is known as the ‘the hand‐in‐bucket’ sign and is pathognomonic for AWP.^1^ Symmetrical involvement of the palms is the most common presentation of AWP, with the soles sometimes involved.^1^ Other atypical sites for AWP include the forehead, heel and dorsa of the fingers, and unilateral involvement can also be seen.^7^, ^8^, ^9^, ^10^, ^11^ Most cases resolve within 20 min of terminating water exposure.^12^ The possible differential diagnoses of AWP are listed in Table 1.

**Table 1:** Possible differential diagnoses for aquagenic wrinkling of the palms.

Feature	AWP	Aquagenic pruritis	Aquagenic urticaria	HPA
Age of onset	Young adulthood	Adulthood	Adolescence or later	Adolescence
Distribution	Symmetrical; palms and soles	Symmetrical; thighs and upper arms	Trunk and upper arms	Symmetrical margins of palms and soles where there is increased pressure and trauma
Clinical features	Exaggerated wrinkling and eruption of white or translucent papules and plaques within minutes of water exposure	Itching within seconds to minutes of coming contact with water; no visible changes to skin during itching	Development of folliculocentric weals within 30 min water exposure; weals can appear in areas not exposed to water	Translucent, yellowish‐white plaques and papules and/or wrinkling of palms upon exposure to water
Associated findings	Pruritus and/or pain	Excoriations and lichenification of the skin due to scratching	Pruritus and sensations of burning or prickling	Sparse, fine hair and atopic diathesis
Inheritance	Sporadic	Sporadic	Sporadic or familial (rare)	Familial
Course	Transient	Transient	Transient	Permanent

AWP, aquagenic wrinkling of the palms; HPA, hereditary papulotranslucent acrokeratoderma.

## Histological findings

Histology can show spongiosis of the stratum corneum, eccrine acrosyringia dilation, crenulation of luminal cells of the secretory eccrine coils, acanthosis, orthohyperkeratosis and increased capillary proliferation around eccrine glands.^1^, ^13^

## Associations with other conditions

AWP is associated with several conditions including CF, hyperhidrosis, RD, marasmus and AD.^3^, ^5^, ^14^

### Cystic fibrosis

The majority of patients with AWP also have CF, but this is not an invariable finding; AWP is seen in 44–80% of patients with CF^15^ and 25% of CF carriers.^16^ The development of AWP in patients and carriers is due to homozygous or heterozygous mutation of the *CFTR* gene encoding the cystic fibrosis transmembrane conductance regulator (CFTR) protein, which is present in eccrine ducts and regulates electrolyte transport. Defective CFTR proteins result in decreased electrolyte reabsorption, thus producing hypertonic sweat, which increases diffusion of liquids into palmar/plantar skin.^17^ In addition, other studies have suggested abnormal expression of aquaporin 5 in the eccrine glands of CF as the cause of the transient oedema in AWP.^18^ It is also suggested that dysfunction in transient receptor potential vanilloid (TRPV)4 channels leads to dysregulation of water transport in the epithelial cells of patients with CF.^6^ Patient should be screened for CF via sweat test or CF gene mutation panel if they have a family history of CF and/or exhibits symptoms of CF, including but not limited to AWP, abdominal disease, reactive airway disease, nasal polyps, sinus drainage, clubbing, diarrhoea, hyperhidrosis and pancreatic insufficiency. Owing to the strong associations between CF and AWP, other studies have suggested the use of brief immersion in water test of the hands for 7 min as an effective screening tool for patients suspected to have CF.^19^ Thorough investigation for CF should be performed in patients with AWP (Table 1).

### Neurological conditions

Associated paraesthesias, such as pain and pruritus, suggest an additional neurological component to AWP. Sheskin *et al*.^20^ noted an absence of physiological wrinkling in patients with nerve injuries. Wilder‐Smith and Chow^21^ hypothesized that diffusion of water into eccrine ducts disturbs electrolyte homeostasis and affects the membrane stability of surrounding nerve fibres, leading to increased vasomotor firing and subsequent vasoconstriction. The increased vasomotor firing results in small muscle fibre contractions and the vasoconstriction itself results in a loss of volume; both of these result in a downward pull on the skin, which causes palmar wrinkling.^22^

## Association with medication

AWP has also been observed in patients taking medications that affect sodium retention in cells. Notably, these include common drugs such as aspirin, selective cyclooxygenase (COX) inhibitors, angiotensin‐converting enzyme (ACE) inhibitors and angiotensin‐receptor blockers. Carder and Weston^23^ reported a case of rofecoxib‐induced AWP and hypothesized that selective COX‐2 inhibition, known to cause increased sodium reabsorption in the kidneys, may have similar effects on keratinocytes. The increased sodium content of keratinocytes may then increase keratin water‐binding capacity and result in exaggerated wrinkling. Khuu *et al*.^11^ reported a case of aspirin‐induced unilateral AWP and hypothesized a similar mechanism, although they noted that aspirin preferentially inhibits COX‐1 over COX‐2. AWP has also been observed in patients taking aminoglycosides, ACE inhibitors and angiotensin‐receptor blockers, either by potentiating sodium retention or affecting osmotic gradient within keratinocytes.^16^

## Treatment

The most common treatments for AWP involve aluminium‐based topicals, with 20% aluminium chloride solution shown to dramatically improve symptoms in patients.^24^ If the patient cannot tolerate the irritating properties of the alcohol solution, a 15% aluminium chloride gel can be used.^2^ Botulinum toxin (BTX)‐A can be used to manage associated hyperhidrosis and autonomic nerve dysfunction and has been recommended as second‐line therapy for patients who do not respond to topical treatments.^1^ Measures that create a barrier to water exposure, including gloves, ammonium lactate creams and petrolatum, have been shown to be ineffective in treating AWP.^25^, ^26^ Other treatment options include 20% salicylic acid, mometasone furoate ointment and 10% urea cream.^27^, ^28^ There are several reports on the use of topical steroids in patients with AWP; however, the benefits of topical corticosteroids in treating AWP are unclear.^29^, ^30^ A list of treatment options is shown in Table 2. Aluminium‐based topicals should be considered first in patients with AWP. If a patient cannot tolerate the adverse effects of the aluminium‐based topicals, the dose should be lowered or the patient switched to a different topical. If symptoms of AWP continue to persist despite trying different topicals, BTX‐A injections can be considered.

**Table 2:** Tests and treatments for aquagenic wrinkling of the palms.

Diagnosis of AWP
Clinical history and physical findings
Brief water immersion test of the palms for 7 min
Skin biopsy
Associated diagnostic tests (for CF)
Genetic test
Sweat test
Treatment options
Aluminium‐based topicals
BTX‐A injections
Salicylic acid
Topical steroids
Urea

AWP, aquagenic wrinkling of the palms; BTX, botulinum toxin; CF, cystic fibrosis.

## Conclusion

AWP is an interesting condition with multiple hypothesized mechanisms and a likely multifactorial aetiology. It is strongly associated with CF, and a diagnosis of AWP should prompt thorough investigations for CF. Beyond CF, the literature also reveals an association with common medications that affect sodium transport. Treatment consists primarily of topical aluminium chloride, though many patients are not bothered by their symptoms and defer any treatment.

**Learning points**

- AWP is a rare phenomenon characterized by transient oedematous white or translucent papules without erythema on the palmar surfaces after contact with water.
- Symmetrical involvement of the palms is the most common presentation of AWP, with the soles sometimes involved.
- Histological findings include spongiosis of the stratum corneum, eccrine acrosyringia dilation, crenulation of luminal cells of the secretory eccrine coils, acanthosis, orthohyperkeratosis and increased capillary proliferation around eccrine glands.
- AWP is strongly associated with CF; 44–80% of patients and 25% of carriers have AWP.
- The development of AWP in patients with CF and CF carriers is due to mutations in the *CFTR* gene, abnormal expression of aquaporin 5 in the eccrine glands, and/or dysfunction in TRPV4 in the epithelial cells of patients with CF.
- A diagnosis of AWP should prompt thorough investigation for CF.

## Conflict of interest

The authors declare that they have no conflicts of interest.

## Funding

None.

## Ethics statement

Ethics approval not applicable. The patient provided informed consent for publication of their case details and images.

## CPD questions

### Learning objective

To gain knowledge on the features of aquagenic wrinkling of the palms, its diagnosis and treatment, and associated conditions.

### Question 1

What inherited disorder is commonly associated with aquagenic wrinkling of the palms (AWP)?

(a) Charcot–Marie–Tooth disease.
(b) Cystic fibrosis.
(c) DiGeorge syndrome.
(d) Huntington disease.
(e) Prader–Willi syndrome.

### Question 2

Which body part does aquagenic wrinkling of the palms (AWP) affect?

(a) Axilla.
(b) Buttocks.
(c) Gums.
(d) Palms.
(e) Sclera.

### Question 3

How long after water immersion does wrinkling of palmar skin occur in patients with aquagenic wrinkling of the palms (AWP)?

(a) 1 minute.
(b) 3 minutes.
(c) 8 minutes.
(d) 9 minutes.
(e) 10 minutes.

### Question 4

If a patient is diagnosed with aquagenic wrinkling of the palms (AWP), thorough investigation should be ordered for which disorder?

(a) Bowel cancer.
(b) Crohn disease (CD).
(c) Cystic fibrosis (CF).
(d) Diabetes.
(e) Haemophilia.

### Question 5

Dysfunction of a particular protein results in decreased electrolyte reabsorption and resultant hypertonic sweat, which increases diffusion of liquids into palmar/plantar skin in patients with cystic fibrosis who have aquagenic wrinkling of the palms (AWP); which protein is this?

(a) Cystic fibrosis conductance regulator protein.
(b) Filaggrin.
(c) Insulin receptor.
(d) Rhodopsin.
(e) Sodium glucose cotransporter.

## Instructions for answering questions

This learning activity is freely available online at http://www.wileyhealthlearning.com/ced

Users are encouraged to

- Read the article in print or online, paying particular attention to the learning points and any author conflict of interest disclosures.
- Reflect on the article.
- Register or login online at http://www.wileyhealthlearning.com/ced and answer the CPD questions.
- Complete the required evaluation component of the activity.

Once the test is passed, you will receive a certificate and the learning activity can be added to your RCP CPD diary as a self‐certified entry.

This activity will be available for CPD credit for 2 years following its publication date. At that time, it will be reviewed and potentially updated and extended for an additional period.

## Data Availability

Not applicable.
